# Chemotherapy-induced nausea and vomiting (CINV) with carboplatin plus pemetrexed or carboplatin plus paclitaxel in patients with lung cancer: a propensity score-matched analysis

**DOI:** 10.1186/s12885-021-07802-y

**Published:** 2021-01-15

**Authors:** Toshinobu Hayashi, Mototsugu Shimokawa, Koichi Matsuo, Hirotoshi Iihara, Kei Kawada, Takafumi Nakano, Takashi Egawa

**Affiliations:** 1 Department of Pharmaceutical and Health Care Management, Faculty of Pharmaceutical Sciences, Fukuoka University, 8-19-1, Nanakuma. Jonan-ku, Fukuoka, 814-0180 Japan; 2 Department of Biostatistics, Yamaguchi University Graduate School of Medicine, 1-1-1, Minamiogushi, Ube, Yamaguchi, 755-8505 Japan; 3 Department of Pharmacy, Fukuoka University Chikushi Hospital, 1-1-1, Zokumyoin, Chikushino, Fukuoka, 818-0067 Japan; 4 Department of Pharmacy, Gifu University Hospital, 1-1, Yanagido, Gifu City, 501-1194 Japan; 5 Department of Pharmacy, Kochi Medical School Hospital, 185-1 Kohasu, Oko town, Nankoku City, Kochi 783-8505 Japan

**Keywords:** Antiemetics, Chemotherapy-induced nausea and vomiting, Emetogenicity, Lung cancer, Classification, Carboplatin

## Abstract

**Background:**

Patients with lung cancer who are treated with carboplatin-based chemotherapy regimens often experience chemotherapy-induced nausea and vomiting (CINV). However, knowledge on the effect of regimen and cofactors on the risk of CINV is limited. This study aimed to analyze and compare the incidence of CINV between lung cancer patients undergoing carboplatin plus pemetrexed (CBDCA+PEM) and those undergoing carboplatin plus paclitaxel (CBDCA+PTX) chemotherapy.

**Methods:**

Pooled data of 240 patients from two prospective observational studies were compared using propensity score matching. Separate multivariate logistic regression analyses were used to identify risk factors for nausea and vomiting following chemotherapy.

**Results:**

Delayed nausea was significantly more common in patients treated with CBDCA+PEM than in those treated with CBDCA+PTX (51.1% vs. 36.2%, *P* = 0.04), but the incidence of vomiting did not significantly differ between the two groups (23.4% vs. 14.9%, *P* = 0.14). The occurrence of CINV peaked on day 4 in the CBDCA+PTX group and on day 5 in the CBDCA+PEM group. Multivariate analysis showed that female sex, younger age, and CBDCA+PEM regimen were independent risk factors for delayed nausea, while female sex was an independent risk factor for delayed vomiting.

**Conclusions:**

The CBDCA + PEM regimen has a higher risk of causing delayed nausea than the CBDCA + PTX regimen, and aggressive antiemetic prophylaxis should be offered to patients treated with CBDCA + PEM.

**Supplementary Information:**

The online version contains supplementary material available at 10.1186/s12885-021-07802-y.

## Background

Chemotherapy-induced nausea and vomiting (CINV) is one of the most dreaded and distressing adverse events in cancer chemotherapy. Antiemetic treatment was markedly improved by the development of antiemetic agents such as 5-hydroxytryptamine-3 receptor antagonists (5-HT3RAs) and neurokinin-1 receptor antagonists (NK1RAs). Further, guidelines for antiemetic therapy are now available including those prepared by the American Society of Clinical Oncology [1], the Multinational Association of Supportive Care in Cancer/European Society of Medical Oncology [2], the National Comprehensive Cancer Network [3], and Japanese Society of Clinical Oncology [4]. The emetogenic classification of antineoplastic agents has also provided a framework for defining antiemetic treatment recommendations in these guidelines. The most widely used classification schema identifies four emetic risk levels (i.e., expected frequency of emesis in the absence of antiemetic prophylaxis): minimal emetic risk, < 10%; low emetic risk, 10–30%; moderate emetic risk, 30–90%; and high emetic risk, > 90% [5]. Despite these improvements, CINV in high emetic-risk chemotherapies remains insufficiently controlled, particularly in the delayed phase.

In the recently updated guidelines [1–4], CBDCA (AUC > 4) was re-classified from a moderate emetic risk group to a high emetic risk group, and a three-drug combination of a 5-HT3RA, NK1RA, and dexamethasone (DEX) was recommended for antiemetic treatment. However, this change was based on the results obtained from patients who received CBDCA as a part of a moderate emetic risk chemotherapy regimen or from studies with a small sample size [6–12]. Although antineoplastic agents or combination regimens can have the same emetic risk category, each agent or combination regimen may have different emetogenicity. However, accurately defining the emetic risk of each agent or regimen remains challenging, and thus information on the emetogenicity of each agent or chemotherapeutic regimen is currently lacking. Precise information on the emetic risk of each agent or regimen will be helpful for the development of effective and individualized antiemetic treatment.

We previously reported that pemetrexed or gemcitabine has higher emetic risk than taxanes (e.g., paclitaxel or docetaxel) [13]. Therefore, we hypothesized that the incidence and the patterns of CINV differ depending on the type of low emetic risk antineoplastic agent that was combined with CBDCA. In this study, we aimed to investigate the risk factors for CINV in patients with lung cancer receiving carboplatin plus pemetrexed (CBDCA+PTX) or carboplatin plus paclitaxel (CBDCA+PEM) and determine the difference in the emetic risk between these two regimens.

## Methods

### Study design

**Fig. 1:**
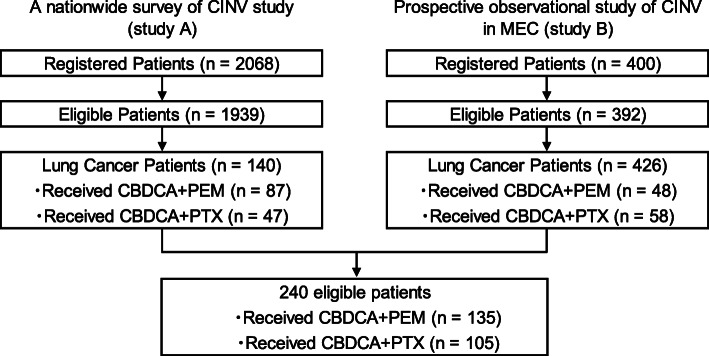
Patient inclusion flowchart. Pooled data from a total of 240 lung cancer patients who received CBDCA+PEM or CBDCA+PTX were selected out of 2468 patients from two prospective observational studies and analyzed

We analyzed pooled data of 240 patients from two multicenter, prospective, observational studies. Individual study results were previously published [14, 15]. The patient selection flowchart is shown in Fig. 1. Both studies included patients scheduled to receive moderately emetic chemotherapy in Japan and were approved by their respective institutional review boards [study A, the institutional review board of Fukuoka University Hospital; study B, the institutional review board of National Cancer Center Hospital East]. Written informed consent was obtained from all participating patients before any related study procedure was initiated. All procedures performed in studies involving human participants were in accordance with the ethical standards of the institutional research committee and with the 1964 Helsinki declaration and its later amendments or comparable ethical standards.

### Data collection

Patients enrolled in these two studies were required to be at least 20 years of age, have solid cancer, and be chemotherapy-naïve. The data source included 7-day diaries that started on the day of chemotherapy. CINV data were based on patient self-reports using their diaries. Eligible patients received two antiemetics (palonosetron or first-generation 5-HT3RAs [i.e., ondansetron, and granisetron] and DEX) or three antiemetics (two antiemetics plus aprepitant), all of which were given within 1 h before their scheduled chemotherapy.

### Statistical analysis

Patient characteristics and the incidence of CINV were summarized using descriptive statistics or contingency tables and were compared using the Student’s *t*-test and Chi-square test. Propensity score matching (PSM) was used to balance the characteristics between the CBDCA+PEM group and the CBDCA+PTX group and to reduce bias. Propensity scores to determine matched pairs between the groups were created using five variables (age, sex, drinking habit, history of motion sickness, and antiemetic prophylaxis) that could potentially influence the occurrence of CINV for patients with lung cancer. The propensity scores were then calculated using a logistic regression model. Patients were matched in a 1:1 ratio using a calliper width of 0.2 of the standard deviation from the propensity score logit.

Additionally, independent risk factors for CINV were evaluated using logistic regression analysis with the backward elimination method. A two-sided *P* value of < 0.05 was considered significant, except for independent risk factors where the significance level was set at *P* < 0.1.

Furthermore, the antiemetic treatment failure curves in each chemotherapeutic regimen group were evaluated using the Kaplan– Meier method and compared between the two groups using the log-rank test. The PSM population was used for all analyses of the occurrence and risk factors for CINV. Sensitivity analysis was conducted to determine whether this conclusion was consistent between the overall cohort and the PSM population. Data from the overall cohort (before PSM matching) and from the PSM population were compared. All statistical analyses were performed using SAS 9.4 (SAS Institute, Cary, NC, USA).

## Results

### Patient characteristics

A total of 240 patients were included in the analysis: 134 (55.8%) and 106 (44.2%) patients from studies A and B, respectively. Overall, 135 (87 and 48 in studies A and B, respectively) received CBDCA+PEM, and 105 (47 and 58 in studies A and B, respectively) received CBDCA+PTX. Their baseline characteristics including age, motion sickness, drinking habits, and antiemetic prophylaxis are shown in Table 1. There were no significant differences between the two groups after the PSM (Table 1).

**Table 1:** Patient characteristics

Characteristics		Before propensity score matching					After propensity score matching				
		CBDCA+PEM		CBDCA+PTX		*p*-value	CBDCA+PEM		CBDCA+PTX		*p*-value
		(*n* = 135)		(*n* = 105)			(*n* = 94)		(n = 94)		
		n	(%)	n	(%)		n	(%)	n	(%)	
Sex	Male	84	(62.2)	89	(84.8)	< 0.001	78	(83.0)	78	(83.0)	1.000
	Female	51	(37.8)	16	(15.2)		16	(17.0)	16	(17.0)	
Age, years	< 65	56	(41.5)	46	(43.8)	0.793	37	(39.4)	43	(45.7)	0.461
	≥65	79	(58.5)	59	(56.2)		57	(60.6)	51	(54.3)	
Motion sickness	No	122	(90.4)	99	(94.3)	0.338	85	(90.4)	89	(94.7)	0.406
	Yes	13	(9.6)	6	(5.7)		9	(9.6)	5	(5.3)	
Drinking habit	No	78	(57.8)	52	(49.5)	0.240	45	(47.9)	45	(47.9)	1.000
	Yes	57	(42.2)	53	(50.5)		49	(52.1)	49	(52.1)	
Number of antiemetics	2	95	(70.4)	91	(86.7)	0.003	80	(85.1)	80	(85.1)	1.000
	3	40	(29.6)	14	(13.3)		14	(14.9)	14	(14.9)	

*CBDCA+PEM* carboplatin+pemetrexed, *CBDCA+PTX* carboplatin+paclitaxel

### Incidence of CINV

**Fig. 2:**
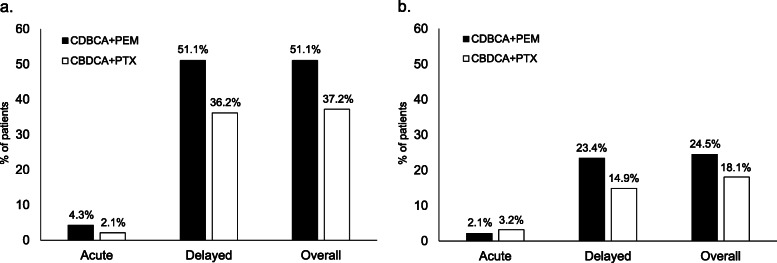
Incidence of nausea and vomiting. The incidence of nausea (**a**) and vomiting (**b**) in the acute phase, delayed phase, and overall treatment period. Black bars denote the CBDCA+PEM group, while white bars indicate the CBDCA+PTX group. The incidence of delayed nausea was significantly higher in the CBDCA+PEM group than that in the CBDCA+PTX group

The incidence of CINV is shown in Fig. 2. The incidence of delayed nausea was significantly higher in the matched CBDCA+PEM group than that in the matched CBDCA+PTX group (51.1% vs. 36.2%, *P* = 0.040). However, there were no significant differences between the matched CBDCA+PEM group and the matched CBDCA+PTX group with respect to the incidence of overall, acute, or delayed vomiting.

### Pattern of CINV incidence

**Fig. 3:**
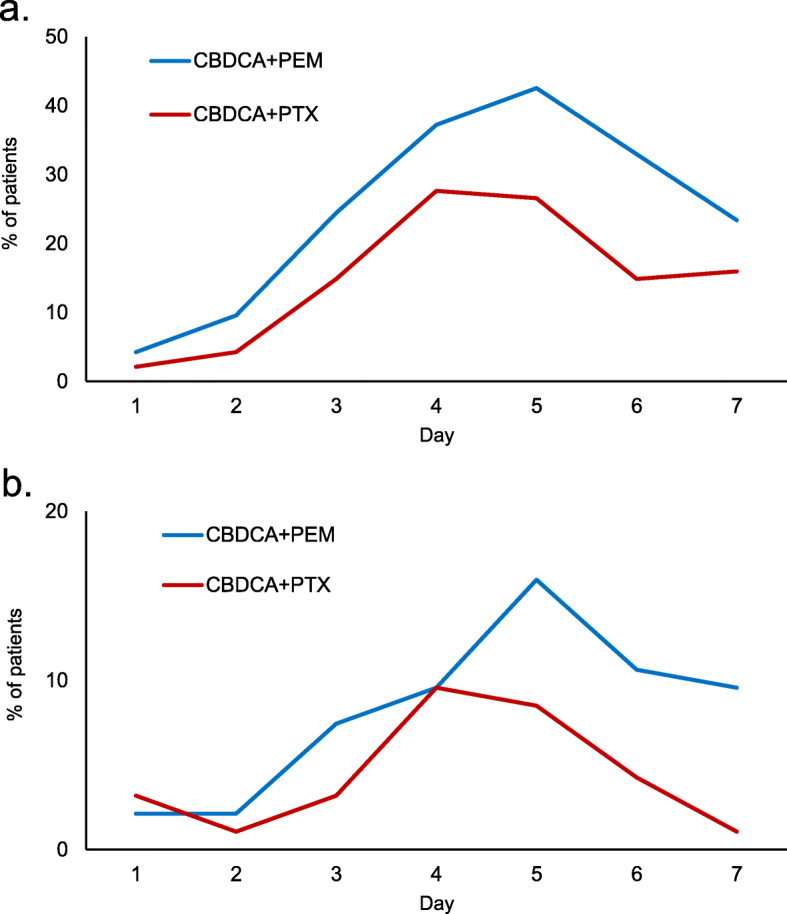
Patterns of CINV occurrence. Occurrence pattern of nausea (**a**) and vomiting (**b**) from day 1 to day 7. The occurrence of nausea and vomiting peaked on days 5 and 4 for the CBDCA+PEM and the CBDCA+PTX groups, respectively. CINV tended to be delayed in the CBDCA+PEM group

**Fig. 4:**
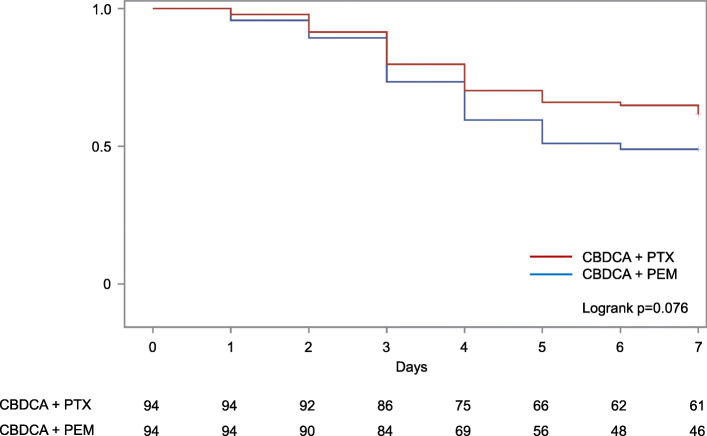
Time to antiemetic treatment failure in nausea. Kaplan–Meier curves of time to nausea event according to each chemotherapeutic regimen showed that there was no statistically significant difference

The incidence of CINV occurring from day 1 to day 7 after chemotherapy is shown in Fig. 3. In both groups, the incidence of nausea on days 1 and 2 was low and gradually increased, peaking on days 4 and 5, followed by a gradual decline. The incidence of vomiting in the CBDCA+PTX group showed a similar pattern, whereas the incidence of vomiting in the CBDCA+PEM group remained high after day 5. Kaplan–Meier curve of time to antiemetic treatment failure (TTF) in nausea is shown in Fig. 4. Kaplan–Meier curves of time to nausea event according to each chemotherapeutic regimen showed that there was no statistically significant difference between the two groups.

### Risk factors for CINV

**Fig. 5:**
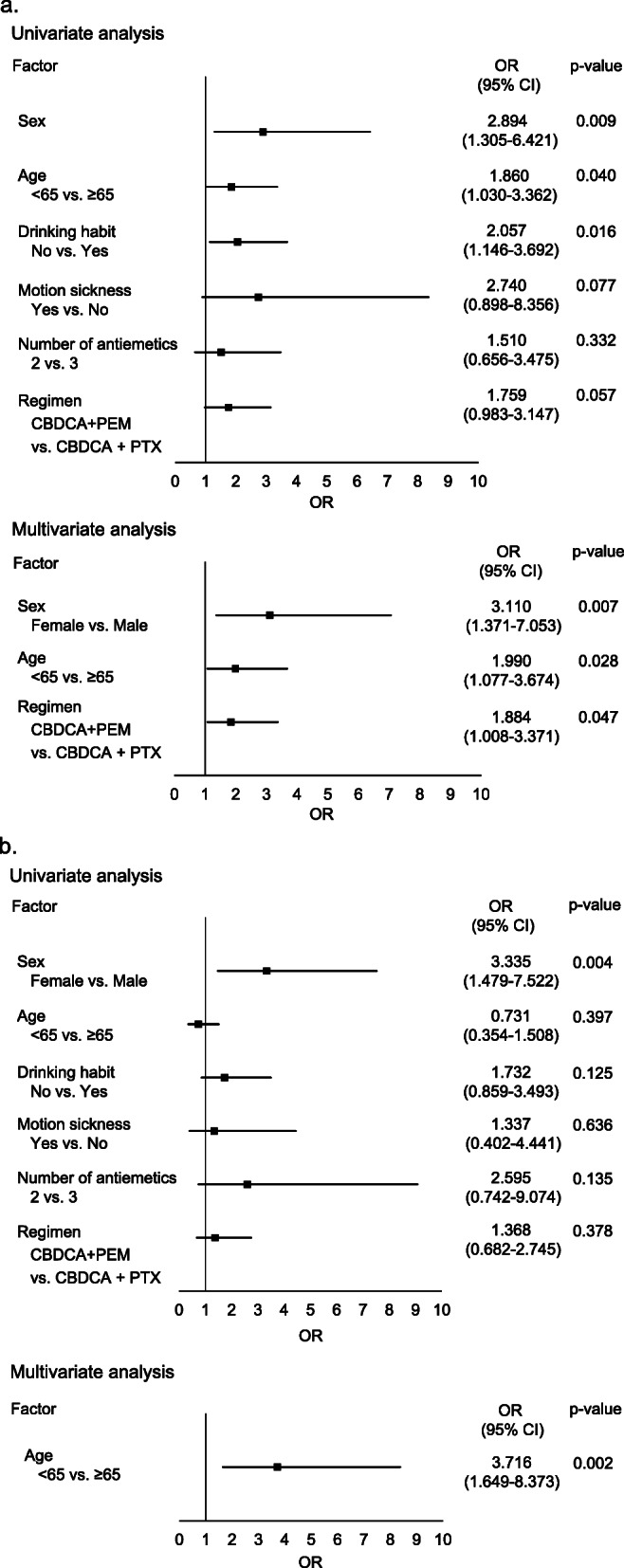
Risk factors for delayed nausea and vomiting. Univariate and multivariate analyses were conducted to identify the risk factors for delayed nausea (**a**) and vomiting (**b**). OR; odds ratio, CI; confidence interval

The results of univariate and multivariate logistic regression analyses of risk factors for delayed CINV are shown in Fig. 5. Sex, age, motion sickness, drinking habits, antiemetic prophylaxis, and chemotherapeutic regimen were analyzed. Female sex, age < 65 years, drinking habit, double antiemetic regimen, and CBDCA+PEM were identified as risk factors for delayed nausea. Meanwhile, female sex and two-antiemetic regimen were identified as risk factors for delayed vomiting.

### Sensitivity analysis

The results of the sensitivity analysis for the patterns in the incidence of CINV and TTF in nausea were consistent across the overall and PSM population (Figs. 3 and 4, Supplementary figures 1 and 2).

## Discussion

The present study demonstrated a higher incidence of CINV in patients who received CBDCA+PEM than in those who received CBDCA+PTX therapy. Further, the CBDCA+PEM regimen was an independent risk factor for nausea in the delayed phase. While acute CINV was well controlled in both groups, the incidence of delayed nausea was significantly higher in the CBDCA+PEM group than that in the CBDCA+PTX group (51.1% vs. 36.2%, *P* = 0.040). Although the incidence of delayed vomiting was not significantly different between the two groups, the incidence of vomiting in the CBDCA+PEM group tended to be high (23.4% vs. 14.9%, *P* = 0.138).

Knowing the CINV incidence pattern is important for determining the optimal antiemetic treatment. In patients who received anthracycline + cyclophosphamide regimens with antiemetic prophylaxis, nausea peaked on day 2 [14]. Meanwhile, in this study, nausea and vomiting peaked on day 5 in patients who received CBDCA regimens with antiemetic prophylaxis. This incidence pattern was similar to that observed for cisplatin [14]. Although there were no significant differences in the time to nausea event (*p* = 0.076), the incidence rate of CINV, patterns of CINV incidence, and TTF suggest that the control of delayed nausea in patients receiving CBDCA+PEM needs to be improved.

Moreover, it is important to identify patients with a high risk of CINV so that appropriate measures to preserve the quality of life and ensure continuity of chemotherapy can be implemented. In this study, logistic regression analysis identified younger age, female sex, and CBDCA+PEM as independent risk factors associated with delayed nausea, and female sex as associated with delayed vomiting. Female sex and young age are well-known risk factors for CINV [16–19]. It is worth noting that the CBDCA+PEM regimen has been identified in this study as an independent risk factor for delayed nausea, along with these well-known patient-related risk factors.

We previously reported that the incidence of CINV was significantly higher in patients receiving pemetrexed or gemcitabine (GEM) than that in patients receiving taxane [13], and consistent results were obtained in this study. The CBDCA+PTX regimen requires premedication of 20 mg DEX, H1-blocker, and H2-blocker for allergy prevention on day 1. Although these agents may enhance the antiemetic effect, they have limited efficacy, as indicated by the incidence and pattern of CINV observed in this study. Therefore, the optimal antiemetic therapy still needs to be determined carefully for each CBDCA-based regimen, even for those in the same emetic risk category.

CINV in patients receiving CBDCA+PTX can be controlled relatively well by two antiemetics [8, 15]. Ito et al. [8] reported that the CBDCA+PEM regimen had relatively high emetic potential, and triple antiemetic therapy with a 5-HT3 receptor antagonist, DEX, and aprepitant may be an effective prophylactic treatment in patients receiving the CBDCA+PEM regimen. However, they evaluated CINV according to complete response (no vomiting and no rescue medication), and the incidence of nausea was unclear. In our study, the incidence of nausea was higher in the CBDCA+PEM group than that in the CBDCA+PTX group at the delayed phase, highlighting the need for improving the control of nausea in the delayed phase. Meanwhile, GEM may also have a higher risk of emesis among low emetic risk chemotherapies [13]. Data from previous clinical trials suggest that CBDCA+GEM may have a higher risk of CINV than CBDCA+PTX [20, 21]. However, we were unable to analyze this case because of the small number of patients who received CBDCA+GEM. In addition, there are no reports directly comparing the incidence of nausea and vomiting between CBDCA+GEM and CBDCA+PTX. It is also unclear whether there is a clinically meaningful difference in terms of emetic risk between CBDCA+PEM and CBDCA+GEM.

The efficacy and safety of 10 mg olanzapine and standard triplet antiemetic therapy were shown in a randomized, double-blind phase III study in patients who received highly emetogenic chemotherapy including anthracycline/cyclophosphamide and cisplatin [22]. Hashimoto et al. [23] reported that 5 mg of olanzapine combined with aprepitant, palonosetron, and dexamethasone provided a significant improvement in delayed CINV. Further, there were no significant differences in the incidence of daytime sleepiness between the olanzapine group and the placebo group among the patients receiving cisplatin-based chemotherapy. Tanaka et al. [24] reported that adding olanzapine to aprepitant, 5-HT3RA, and DEX improved CINV control in patients receiving CBDCA-based chemotherapy. Adding olanzapine to standard triplet antiemetic therapy is considered a promising option to improve control of delayed nausea in patients receiving CBDCA+PEM.

This study has some limitations. First, it was not a blinded, randomized control trial. Second, some risk factors of CINV such as smoking habits and morning sickness could not be analyzed. Finally, the results of this study were obtained from a Japanese population, and while the results may be applicable to other Asian populations, further research is needed to verify the generalizability of these results to other races. Despite these limitations, the findings described the emetogenicity of and risk factors for CBDCA-based regimens in routine clinical practice, rather than in a controlled trial, and therefore might reflect the real-world conditions. Further, the use of data with a sufficient number of events from two prospective observational studies and PSM enabled high accuracy and robustness of the results.

## Conclusion

The CBDCA + PEM regimen had a higher risk of causing nausea than the CBDCA + PTX regimen. The optimal antiemetic therapy for each regimen in CBDCA-based chemotherapy should be carefully chosen because delayed CINV, especially nausea, is still insufficiently controlled in some patients who receive the CDBCA+PEM regimen. Patients who are female, aged < 65 years, and receiving CBDCA+PEM have a high risk of CINV, and thus additional antiemetics for delayed CINV (e.g., olanzapine) should be considered for these patients.

## Supplementary Information

**Additional file 1: Supplementary figure 1.** Patterns of CINV occurrence in overall population. Occurrence pattern of nausea (a) and vomiting (b) from day 1 to day 7. The patterns in the incidence of CINV was consistent across the overall and PSM population.

**Additional file 2: Supplementary figure 2.** TTF of nausea in overall population. Kaplan–Meier curves of time to nausea event according to each chemotherapeutic regimen showed that there was statistically significant difference between the two groups.

## Data Availability

Availability of data and material: The data that support the findings of this study are available from both study groups of study A and study B but restrictions apply to the availability of these data, which were used under license for the current study, and therefore, the data are not publicly available. However, data are available from the authors upon reasonable request and with permission of both study groups of study A and study B.

## Abbreviations

CINV: Chemotherapy-induced nausea and vomiting
CBDCA+PEM: Cancer patients undergoing carboplatin plus pemetrexed
CBDCA+PTX: Paclitaxel
5-HT3RAs: 5-hydroxytryptamine-3 receptor antagonists
NK1RAs: Neurokinin-1 receptor antagonists
DEX: Dexamethasone
