# Supplementary figures and images for: Chemotherapy-induced nausea and vomiting (CINV) with carboplatin plus pemetrexed or carboplatin plus paclitaxel in patients with lung cancer: a propensity score-matched analysis
Source: BMC Cancer. 2021 Jan 15;21:74. doi: 10.1186/s12885-021-07802-y (PMC7811213; doi:10.1186/s12885-021-07802-y)

## Slide 1
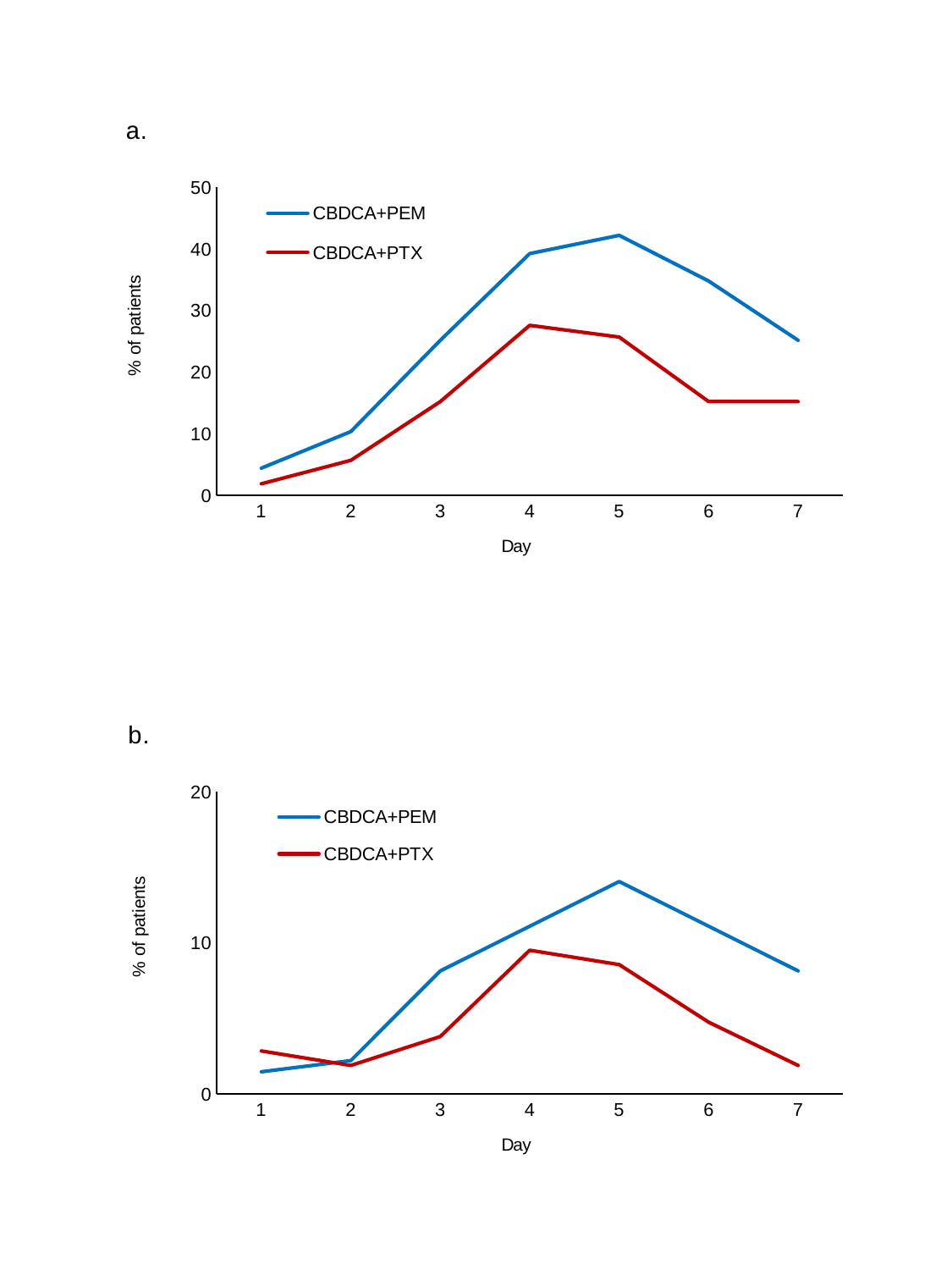

### Chart: a.
| Category | CBDCA+PEM | CBDCA+PTX |
|---|---|---|
| 1 | 4.44 | 1.9 |
| 2 | 10.37 | 5.71 |
| 3 | 25.19 | 15.24 |
| 4 | 39.26 | 27.62 |
| 5 | 42.22 | 25.71 |
| 6 | 34.81 | 15.24 |
| 7 | 25.19 | 15.24 |
### Chart: b.
| Category | CBDCA+PEM | CBDCA+PTX |
|---|---|---|
| 1 | 1.48 | 2.86 |
| 2 | 2.22 | 1.9 |
| 3 | 8.15 | 3.81 |
| 4 | 11.11 | 9.52 |
| 5 | 14.07 | 8.57 |
| 6 | 11.11 | 4.76 |
| 7 | 8.15 | 1.9 |

Supplement: Supplementary file 1 — Additional file 1: Supplementary figure 1. Patterns of CINV occurrence in overall population. Occurrence pattern of nausea (a) and vomiting (b) from day 1 to day 7. The patterns in the incidence of CINV was consistent across the overall and PSM population. [file 12885_2021_7802_MOESM1_ESM.pptx]

## Slide 1
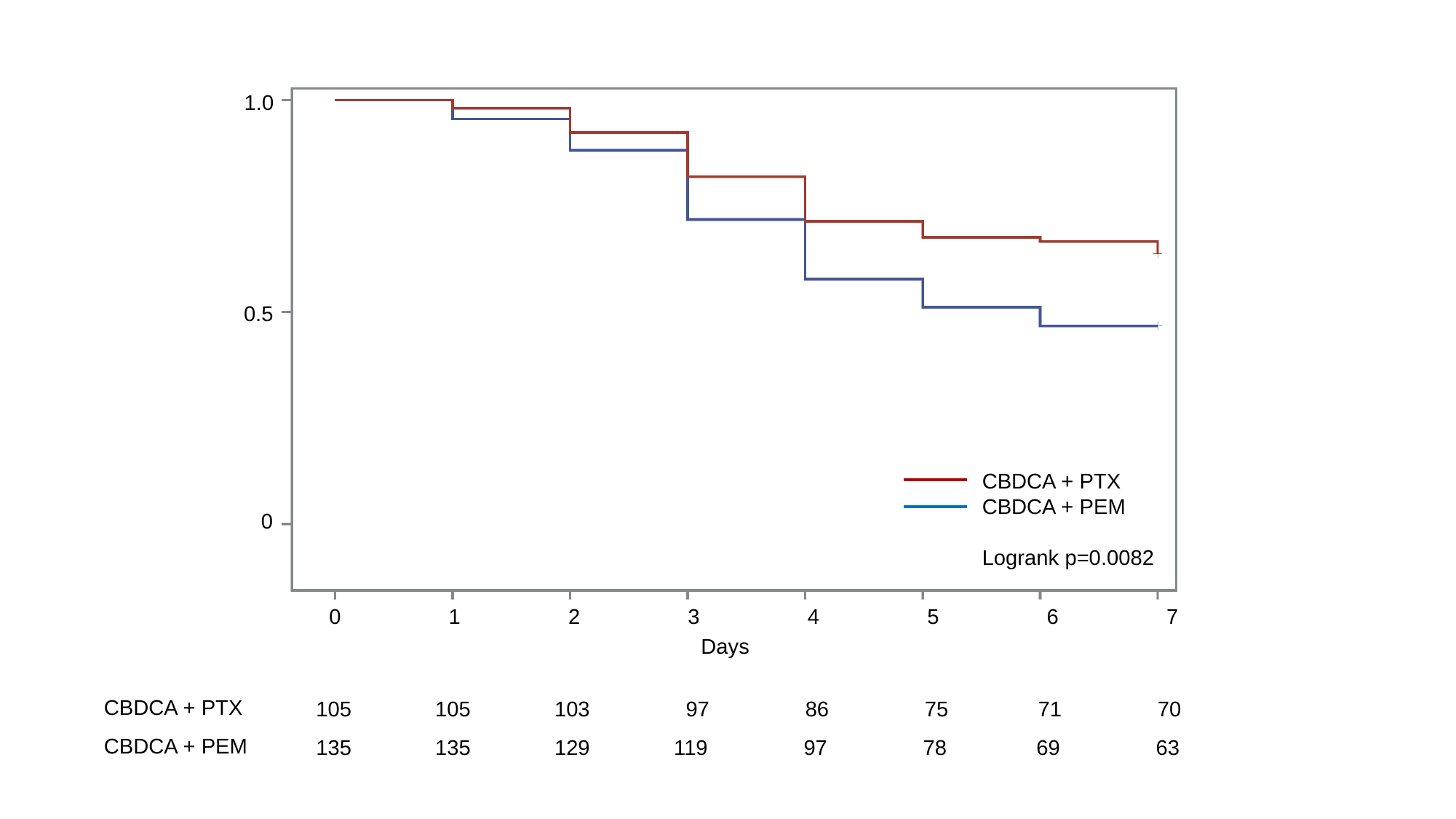

1.0
0.5
CBDCA + PTX
CBDCA + PEM
Logrank p=0.0082
0
0 1 2 3 4 5 6 7
Days
CBDCA + PTX
CBDCA + PEM
105 105 103 97 86 75 71 70
135 135 129 119 97 78 69 63

Supplement: Supplementary file 2 — Additional file 2: Supplementary figure 2. TTF of nausea in overall population. Kaplan–Meier curves of time to nausea event according to each chemotherapeutic regimen showed that there was statistically significant difference between the two groups. [file 12885_2021_7802_MOESM2_ESM.pptx]
